# Mr. Thelwall, on Cutting the Bridle of the Tongue

**Published:** 1805-09-01

**Authors:** John Thelwall

**Affiliations:** Brownlow Hill, Liverpool


### *To the Editors of the Medical and Physical Journal.*

Gentlemen,

THE professional interest which I necessarily feel in every thing connected with the economy of the organs of voice and enunciation, has occasioned me to peruse, with particular with particular satisfaction, the remarks of Dr. Denman, in the last number of your miscellany, on the rash and mischievous practice of cutting, during the earliest season of infancy, "what is called the bridle of the tongue." I was not, indeed, aware that consequences so fatal as those which gave rise to Dr. D's communication were likely to ensue; but facts enough had fallen under my observation to convince me—that the operation was generally unnecessary, and, so far as related to *the particular object of my enquiry*, more frequently injurious than beneficial; and (in my Lectures on the Causes, Prevention, and Cure of Impediments of Speech) I have, accordingly, not been sparing in my warnings and exhortations against the officiousness of ignorant nurses, in this particular.

Permit me, however, to observe that, as far as relates to the power of distinct utterance, Dr. D. perhaps, expresses himself rather too generally, when he suggests—"that the frænum *never* requires cutting." In early infancy, indeed, I can readily admit that it never does; for, if medical gentlemen will answer for the *fact* so far as relates to the operation of *sucking*, I shall not scruple to affirm—that, for *elocutionary purposes*, it can never be advisable to apply the scissars before the *twelfth* or *fourteenth* year;—that is to say, before sufficient time has been allowed for the experiment, whether the natural action of the organs, in the process of enunciative effort and cultivation, will not stretch so far the *constricted* or *protrusive* frænum "as to allow all the necessary and proper motions of the tongue." I wish it also to be understood, as my decisive opinion, that if, in the mean time, the organs of enunciation *were judiciously educated*, with a marked attention to the efforts for the formation of the *lingual* elements, the progressive elongation which would inevitably ensue, would be, almost universally, adequate to the object in contemplation. But I am necessitated to declare, that there are some instances in which the degrees of effort and attention *actually applied*, have not been sufficient to produce the elongation of frænum and liberty of motion which perfect enunciation requires.

In a single class of private pupils (from the select Academy of the Rev. Mr. Inchbald of Doncaster) were four different cases, every one of which may, in some way or other, be regarded as illustrative of our present subject.

One of these young gentlemen had, in reality, suffered by the officiousness of which Dr. D. and myself, alike complain; so that the tongue, not being sufficiently restricted stricted in its position, had a tendency to advance and coil up in the mouth; and, though not absolutely intractible to volition, required a much more guarded and precise attention to the regulation of its motions, than is requisite in ordinary cases. His brother (whose original conformation had, at least, been equally unfavourable,—and whose mouth exhibited such a protrusive duplication and reduplication of the frænum as I have seldom witnessed) had, fortunately, escaped the malady of a like redress; and the preternatural ligatures had so elongated themselves, as to leave him in possession of powers of utterance—much more likely to be envied for their impressiveness, than censured for imbecility.

The other two, who were brothers also, had not been equally successful. In the younger of these, a sort of lisp was produced, from the almost utter impossibility of so completely removing the entire edge of the tongue from contact with the lower teeth, as to admit the complete percussion of the sharper sounds; while, in the elder, the stricture was so obstinate as, not merely "to form a small indentation at the extremity," but even to divide the tongue, in its attempts to advance beyond the teeth, into two thick lobes, which interdicted the complete formation of the element *th*, and imparted a sort of thickness to the whole enunciation. In both these cases, after reiterating every experiment, which, in other instances, I had found efficacious in subduing similar impediments, I was obliged to invite the assistance of the surgeon; whose operation, most assuredly, was considerably assistant to the efficacy of my instructions.

There is, also, another observation in Dr. Denman's communication, which may, perhaps, be liable to some misapprehension; the statement, I mean, that "what is called speaking thick, pleno ore, is occasioned by cutting the frænum." In its present unqualified shape, this position is, certainly, much too general; for though the officiousness of nurses and gossips, in this particular, is *one* of the primary causes of such impediment, yet does not the defect, on the one hand, *irremediably* ensue, nor, on the other, does thickness of enunciation necessarily suppose any such peculiarity or injury in the physical state of the organs; two propositions sufficiently illustrated in the phenomena and successful treatment of the cases above described. In short, speaking thick does sometimes, undoubtedly, arise from too great *laxity*, and sometimes from too rigid restriction of the apex of the tongue; but, in general general, like almost every other impediment, it will be found to originate in habitual sluggishness, or evil imitation; and the tongue of the thickest mumbler will frequently be found as perfect in its structure and capabilities as that of the most accomplished elocutionist.

While I was cultivating my little farm in Wales, before I had ever thought of taking up my present profession, an instance of this presented itself to my observance, which made a deep impression on my mind. Three children of Mr. Griffiths, a hatter in Brecknock, had contracted such a habit of coiling up the tongue, as rendered their speech almost unintelligible. Their parents had, accordingly, conceived that the boys had a natural impediment,—or, as they expressed it, "that their mouths were not made like other people's mouths." From this impression, it is probable that the lads would have been permitted to grow up in the habit of negligent utterance, till it had ripened into inveterate impediment, if the accident of my going into the shop to furnish myself with an article I wanted, had not brought me acquainted with the circumstance. Half an hour's attention, however, and the imposition of a very acceptable task (the reiterated pronunciation of a short, ridiculous sentence) enabled me to put them into a train of as intelligible utterance as any of the people by whom they were surrounded.

To conclude.—Let it not be supposed, upon one hand, that wherever there is a thickness of utterance, or indistinct enunciation, there must necessarily be any defect in the physical state of the organs; or, on the other,—that, even where the frænum has been injudiciously separated, or the tongue, from any other circumstance, is more loosely situated in the mouth than usual, that a mumbling fulness must inevitably exist. *Natural impediments*, (properly understood) are, indeed, exceedingly rare. Deafness and mental imbecility excepted,—they can only originate from extreme obstinacy of stricture in the frænum, from hare-lip, from malconformation of the jaw, or from fissure of the roof and obliteration of the uvula; and even of these, fortunately, there is not one which is beyond the reach and medicature of human art; or which may not yield to the co-operative influence of elocutionary and physiological science.

Your's, respectfully,

JOHN THELWALL.

*Brownlow Hill, Liverpool, August* 6, 1805.

